# Effects of hyperinsulinemia on pancreatic cancer development and the immune microenvironment revealed through single-cell transcriptomics

**DOI:** 10.1186/s40170-022-00282-z

**Published:** 2022-02-21

**Authors:** Anni M. Y. Zhang, Ken H. Chu, Brian F. Daly, Titine Ruiter, Yan Dou, Jenny C. C. Yang, Twan J. J. de Winter, Justin Chhuor, Su Wang, Stephane Flibotte, Yiwei Bernie Zhao, Xiaoke Hu, Hong Li, Elizabeth J. Rideout, David F. Schaeffer, James D. Johnson, Janel L. Kopp

**Affiliations:** 1 Department of Cellular and Physiological Sciences, Life Sciences Institute, University of British Columbia, Vancouver, Canada; 2 Life Sciences Institute Bioinformatics Core Facility, University of British Columbia, Vancouver, Canada; 3 Biomedical Research Centre, School of Biomedical Engineering, University of British Columbia, Vancouver, Canada; 4 Department of Pathology and Laboratory and Medicine, University of British Columbia, Vancouver, British Columbia Canada

**Keywords:** Pancreatic intraepithelial neoplasia, Pancreatic ductal adenocarcinoma, Insulin, Obesity, Type 2 diabetes, Single-cell RNA sequencing, Genetically engineered mice

## Abstract

**Background:**

Hyperinsulinemia is independently associated with increased risk and mortality of pancreatic cancer. We recently reported that genetically reduced insulin production resulted in ~ 50% suppression of pancreatic intraepithelial neoplasia (PanIN) precancerous lesions in mice. However, only female mice remained normoglycemic, and only the gene dosage of the rodent-specific *Ins1* alleles was tested in our previous model. Moreover, we did not delve into the molecular and cellular mechanisms associated with modulating hyperinsulinemia.

**Methods:**

We studied how reduced *Ins2* gene dosage affects PanIN lesion development in both male and female *Ptf1a*^*Cre*ER^;*Kras*^LSL-G12D^ mice lacking the rodent-specific *Ins1* gene (*Ins1*^-/-^). We generated control mice having two alleles of the wild-type *Ins2* gene (*Ptf1a*^*Cre*ER^;*Kras*^LSL-G12D^;*Ins1*^-/-^;*Ins2*^+/+^) and experimental mice having one allele of *Ins2* gene (*Ptf1a*^*Cre*ER^;*Kras*^LSL-G12D^;*Ins1*^-/-^;*Ins2*^+/-^). We then performed thorough histopathological analyses and single-cell transcriptomics for both genotypes and sexes.

**Results:**

High-fat diet–induced hyperinsulinemia was transiently or modestly reduced in female and male mice, respectively, with only one allele of *Ins2*. This occurred without dramatically affecting glucose tolerance. Genetic reduction of insulin production resulted in mice with a tendency for less PanIN and acinar-to-ductal metaplasia (ADM) lesions. Using single-cell transcriptomics, we found hyperinsulinemia affected multiple cell types in the pancreas, with the most statistically significant effects on local immune cell types that were highly represented in our sampled cell population. Specifically, hyperinsulinemia modulated pathways associated with protein translation, MAPK-ERK signaling, and PI3K-AKT signaling, which were changed in epithelial cells and subsets of immune cells.

**Conclusions:**

These data suggest a potential role for the immune microenvironment in hyperinsulinemia-driven PanIN development. Together with our previous work, we propose that mild suppression of insulin levels may be useful in preventing pancreatic cancer by acting on multiple cell types.

**Supplementary Information:**

The online version contains supplementary material available at 10.1186/s40170-022-00282-z.

## Introduction

Pancreatic cancer, specifically pancreatic ductal adenocarcinoma (PDAC), is projected to become the 2nd leading cause of cancer death in the next decade. Representing an estimated 2.5% of all new cancer cases, PDAC has a 5-year survival rate of only 10% [1]. Smoking, pancreatitis, family history, obesity, and type 2 diabetes are risk factors for PDAC [2–6]. Obesity is predicted to overtake smoking and become the leading preventable cause of cancer [7], so efforts to understand the roles of diet and lifestyle in cancer risk and prevention strategies are expanding, as are efforts to determine the underlying pathophysiological mechanisms that mediate obesity-driven risk. Obesity and type 2 diabetes are usually accompanied by metabolic disorders like hyperinsulinemia, hyperglycemia, increased inflammation, and dyslipidemia, each of which are candidate factors that may contribute to the associated increase of cancer morbidity and mortality [8–12].

Hyperinsulinemia can be defined as excess circulating insulin beyond what is required for maintaining glucose homeostasis [12]. Excess insulin is associated with an increased risk of cancer death that can be independent of obesity [13]. Hyperinsulinemia is also associated with increased incidence of PDAC and increased cancer mortality rate [14–18]. Complementing these epidemiological studies, our recent *in vivo* animal study showed that hyperinsulinemia plays a causal role in PDAC initiation in the context of a known hyperinsulinemia-promoting high-fat diet (HFD) [19]. Specifically, we found that *Ptf1a*^*Cre*ER^;*Kras*^LSL-G12D^ female mice, a commonly used PDAC mouse model, with ~ 50% reduction in fasting insulin (*Ins1*^+/-^;*Ins2*^-/-^ compared to *Ins1*^+/+^;*Ins2*^-/-^), but no difference in glucose homeostasis, had a ~ 50% reduction in PanIN lesions and fibrogenesis. While we were unable to assess the effects of reduced insulin gene dose in males using this model, as *Ptf1a*^*Cre*ER^;*Kras*^LSL-G12D^;*Ins1*^+/-^;*Ins2*^-/-^ males developed hyperglycemia, these studies were the first to directly demonstrate that endogenous hyperinsulinemia contributes to development of any cancer [19].

The parental imprinting, gene structure and tissue distribution of murine *Ins2* gene is similar to human *INS* gene [20–23]. It is therefore crucial to determine if PanIN initiation would also be affected by reducing *Ins2* gene dosage in *Ptf1a*^*Cre*ER^;*Kras*^LSL-G12D^ mice. Moreover, *Ins1* contributes to only ~ 1/3 of secreted insulin; this means that *Ins1*^+/-^;*Ins2*^-/-^ mice have the lowest amount of insulin compatible with survival, with only female mice remaining consistently normoglycemic in modern mouse housing facilities. In the current study, we addressed the effect of hyperinsulinemia on PDAC development in both sexes by generating *Ptf1a*^*Cre*ER^;*Kras*^LSL-G12D^ mice with the *Ins1*^-/-^;*Ins2*^+/+^ or *Ins1*^-/-^;*Ins2*^+/-^ genotypes. Comparing *Ins1*^-/-^;*Ins2*^+/-^ experimental mice to *Ins1*^-/-^;*Ins2*^+/+^ controls allowed us to compare males and females side-by-side. The current study also provided an opportunity to examine pancreatic single-cell transcriptomes and gain insights into the molecular mechanisms involved in the complex cellular landscape of PDAC initiation under hyperinsulinemic conditions.

## Experimental procedures

### Mice

University of British Columbia Animal Care Committee in accordance with Canadian Council for Animal Care guidelines approved all animal experiments. Mice were kept at the University of British Columbia Modified Barrier Facility (MBF) as previously described in detail [19]. *Ptf1a*^CreER/WT^, *Kras*^LSL-G12D/WT^, *Ins1*^-/-^, and *Ins2*^-/-^ mice have been previously described [19, 22, 24–27]. *Ptf1a*^*Cre*ER/WT^;*Kras*^LSL-G12D/WT^;*Ins1*^-/-^;*Ins2*^+/+^ mice were bred with *Ptf1a*^WT/WT^;*Ins1*^-/-^;*Ins2*^+/-^ mice to generate background-matched control mice (*Ptf1a*^CreER/WT^;*Kras*^LSL-G12D/WT^;*Ins1*^-/-^;*Ins2*^+/+^ mice), and experimental mice (*Ptf1a*^CreER/WT^;*Kras*^LSL-G12D/WT^;*Ins1*^-/-^;*Ins2*^+/-^ mice). The resulting litters were weaned to a high-fat diet with 60% fat (Research Diets D12492; Research Diets). At 4 weeks of age, mice were subcutaneously injected with tamoxifen (20 mg/mL in corn oil, Sigma- Aldrich) at 5 mg tamoxifen/40 g body mass for three consecutive days. At 57 weeks of age, mice were euthanized for histopathological analysis and single-cell transcriptomics analysis. The *Kras*^LSL-G12D/WT^ mice (C57BL/6), *Ptf1a*^CreER/WT^ mice (C57BL/6), and *Ins1*^-/-^ and *Ins2*^-/-^ mice (C57BL/6) were obtained as previously described [19].

### Glucose homeostasis and plasma amylase assessment

Mouse body weight, fasting glucose, and fasting insulin were measured after 4 h of fasting in fresh, clean cages according to standard methods described previously [19]. Body weight and fasting glucose were measured every 4 weeks, and fasting insulin was measured every 3 months. At 52 weeks of age, glucose-stimulated (2 g/kg) insulin secretion, intraperitoneal glucose tolerance test (2 g/kg), and insulin tolerance test (0.75 U/kg, Eli Lilly, USA) were performed as previously described [22, 24, 25]. At 57 weeks of age, blood was collected from mice after 4 h of fasting, and blood samples were centrifuged at 4 °C, at 10,621 relative centrifugal force (rcf) for 10 min to collect blood serum. Fasting amylase levels were measured with an amylase activity assay (MAK009-1KT, MilliporeSigma, MA, USA) according to the manufacturer's instructions.

### Histological, morphological, and immunohistochemical analysis

At 57 weeks of age, mice were euthanized and extracted pancreata were fixed in 4% paraformaldehyde for 24 h followed by paraffin embedding. Seven-micron paraffin sections were collected from every embedded mouse pancreas for a total of 60 sections. Evenly spaced sections were hematoxylin and eosin (H&E) stained and scanned as previously described [19, 28]. Histopathological analysis was conducted by Anni Zhang in a de-identified manner and verified by Janel Kopp and/or David Schaeffer. For each mouse pancreas, the PanIN area, ADM area, and adipocyte area were analyzed on 3 H&E-stained sections, which were 140 μm away from each other. The total pancreatic area, PanIN area, and ADM area were measured as described [19]. For the adipocyte-occupied area, the pancreatic adipose tissue was selected and quantified using the Adobe Photoshop 2020 magic wand tool and measurement log function. The whole mouse pancreas area was selected by using the magnetic lasso and magic wand tools, followed by the measurement log function in Adobe Photoshop 2020. Immunohistochemical (IHC) staining was performed and scanned according to previously published methods [19, 28]. The primary and secondary antibodies used for IHC staining are listed in Table S1. For Cd20- and Erk-positive area measurements, Adobe Photoshop 2020 Black & White function was used to filter and highlight the brown area (blue filter). The percent of Cd20-positive or Erk-positive area per pancreatic section was measured as previously described [19]. Macrophage infiltration was quantified by using the positive pixel count tool in Aperio ImageScope software (Leica Biosystems). In short, mouse formalin fixed paraffin embedded pancreatic sections were first stained for F4/80 antigen to visualize macrophages. Pancreatic pathology areas were then analyzed in each pancreatic section for ADM, PanIN, and PDAC. The infiltration density (%) of F4/80^+^ macrophages in each pathology category was expressed as the pixel area occupied by the DAB staining (F4/80^+^) normalized to the pixel area occupied by the DAB staining plus the hematoxylin staining (all nuclei). The infiltration density for each pathology category is expressed as the average infiltration density of a specific type of pathology for the entire pancreatic section per mouse.

### Tissue dissociation and single-cell sorting

Six *Ptf1a*^*Cre*ER^;*Kras*^LSL-G12D^;*Ins1*^-/-^;*Ins2*^+/+^ mice pancreata and six *Ptf1a*^*Cre*ER^;*Kras*^LSL-G12D^;*Ins1*^-/-^;*Ins2*^+/-^ mice pancreata, evenly divided by sex, were dissociated for single-cell RNA sequencing (scRNAseq) . Briefly, freshly dissected pancreata were washed with HBSS (Corning, 21-021-CV) and minced into ~ 1 mm pieces using sharp dissection scissors. Pieces were transferred into 15 ml conical tubes and centrifuged at 720 rcf for 2 min. The supernatant was discarded, and tissue was resuspended in 5 ml of ice-cold HBSS containing 0.4 mg/ml collagenase P (Roche, #11213857001) and 10 mg/ml DNase I (Roche, #11284932001). Tissue samples were incubated in a 37 °C water bath for 10–18 min, and tubes were gently shaken with marbles. After the dissociation, 10 ml HBSS with 5% FBS (ThermoFisher Scientific, #A3160701) was added to samples, and samples were centrifuged at 720*g* for 2 min. Supernatants were discarded, and samples were washed 3 times with 10 ml HBSS with 5% FBS. Samples were filtered through a 100-μM cell strainer then washed with HBSS with 5% FBS into a 50 ml conical tube. Strainers were washed with 10 ml HBSS with 5% FBS to collect the cells that remained on the strainer. Samples were centrifuged at 180*g* for 2 min to collect the dissociated cells. The resuspended samples were stained with 0.05 μg/ml Hoechst 33342 (Invitrogen, #H3570) and 0.5 μg/ml propidium iodide (Sigma, #P4864) and were sorted for live cells using BD LSR II Flow Cytometer.

### Single-cell transcriptomic data processing, quality control, and analysis

The single cell libraries were prepared with Chromium Single Cell 3’ Reagent Kits V3 (10X genomics, Pleasanton, CS, USA) according to the manufacturer instructions, and the libraries were sequenced on a Nextseq500 (Illumina). The Cell Ranger pipeline (10X genomics, CS, USA) was used for demultiplexing (cellranger mkfastq) and alignment (cellranger count). Ambient RNA contamination artifacts were resolved using the R package SoupX (https://github.com/constantAmateur/SoupX). Cleaned gene-cell matrices were loaded into the R package Seurat 3.0 and filtered to remove cells with unique feature counts over 6000 or less than 200. Cells that had > 20% mitochondrial gene counts or genes that were expressed by fewer than 3 cells were also removed. Using Seurat 3.2.1 [29, 30], the filtered gene–cell matrices from each mouse were integrated and clustered in uniform manifold approximation and projection (UMAP) space using default settings with resolution of 0.1. The typical marker genes (like *Prss2*, *Krt19*, *Col1a1*, and *Ms4a1*) that are commonly used in single-cell transcriptomic analysis of the pancreas were used for identifying clusters [31–33]. When the cluster identity could not be determined, Seurat 3.2.1 FindConservedMarkers function was used to find the top 50 genes that are conserved markers irrespective of the genotype. This gene list was then uploaded to Enrichr (https://maayanlab.cloud/Enrichr/), and we used similarities to the enriched gene expression profiles of different cell types identified by Enrichr to determine the cellular identities for the latter clusters [34].

### Differential gene expression analysis, pathway enrichment analysis, and visualization

A differential gene expression was analyzed between two mouse genotypes (*Ptf1a*^*Cre*ER^; *Kras*^LSL-G12D^;*Ins1*^-/-^;*Ins2*^+/-^ vs *Ptf1a*^*Cre*ER^;*Kras*^LSL-G12D^;*Ins1*^-/-^;*Ins2*^+/+^) to identify upregulated and downregulated genes in each cell type using Seurat 3.2.1 FindMarkers function with default settings [29, 30]]. For the differentially expressed gene lists, pathway enrichment analyses were performed by g:profiler (https://biit.cs.ut.ee/gprofiler/) using the Reactome database (http://www.reactome.org) based on the Reimand *et al*. published protocol [35]. Enriched pathways were visualized using the R package pheatmap 1.0.12. The single-cell expression of some immune checkpoint receptors and ligand genes in each cell type was presented using Seurat 3.2.1 DoHeatmap function [29, 30]. Violin plots were used to visualize single-cell expression distributions for markers of macrophage subtypes and fibroblast subtypes.

### Statistical analysis

**Fig. 1:**
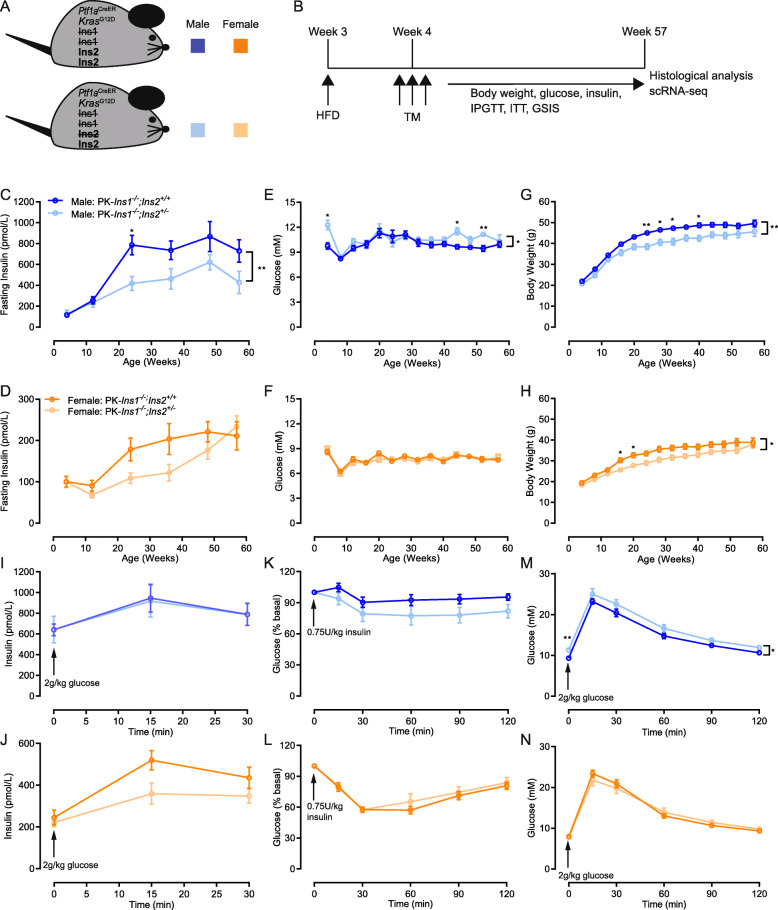
Mice with reduced insulin gene dosage have reduced fasting insulin levels and body weight. **A** Schematic describing a mouse model designed to test the role of insulin on HFD-accelerated PDAC initiation. On the background of *Ptf1a*^CreER^-induced *Kras*^G12D^ pancreatic cancer model (PK), we compared experimental mice with 1 null allele of *Ins2* and control mice with 2 null alleles of *Ins2*, all in the absence of *Ins1* (*Ins1*^*-/-*^) to prevent compensation. **B** Three-week-old PK-*Ins1*^-/-^;*Ins2*^+/+^ control and PK-*Ins1*^-/-^;*Ins2*^+/-^ experimental mice were weaned to a high-fat diet (HFD) and were injected for 3 consecutive days with tamoxifen (TM) beginning at 4 weeks. After repeated physiological measures over the course of a year, the mice were euthanized at 57 weeks of age for histological analysis and scRNA-seq. **C**–**D** Fasting insulin levels in male and female PK-*Ins1*^-/-^;*Ins2*^+/+^ and PK-*Ins1*^-/-^;*Ins2*^+/-^ mice measured over 1 year (*n* = 18–29). **E–F** Fasting glucose levels in male and female PK-*Ins1*^-/-^;*Ins2*^+/+^ and PK-*Ins1*^-/-^;*Ins2*^+/-^ mice measured over 1 year (*n* = 18–29). G–H Body weight in male and female PK-*Ins1*^-/-^;*Ins2*^+/+^ and PK-*Ins1*^-/-^;*Ins2*^+/-^ mice measured over 1 year (*n* = 18–29). **I–J** Glucose-stimulated insulin release in 52-week-old male and female mice (*n* = 17–30). **K–L** Blood glucose response to intraperitoneal delivery of an insulin analog in 52-week-old male and female PK-*Ins1*^-/-^;*Ins2*^+/+^ and PK-*Ins1*^-/-^;*Ins2*^+/-^ mice (*n* = 10–29). **M–N** Blood glucose response to intraperitoneal delivery of glucose in 52-week-old male and female PK-*Ins1*^-/-^;*Ins2*^+/+^ and PK-*Ins1*^-/-^;*Ins2*^+/-^ mice (*n* = 16–29). *p < 0.05 and **p < 0.01. Values are shown as mean ± SEM

Statistical parameters including the sample size *n* (number of animals), mean ± standard error of the mean (SEM), and statistical significance are reported in the figure legends and figures. GraphPad Prism 9.0.0 was used for statistical analysis. Mixed-effects analysis was performed for glucose homeostasis assessments (Fig. 1). When the samples passed a normality test, the two-tailed student’s t test was used; while nonparametric statistics (Mann–Whitney test) was used for data of non-normal distribution (Shapiro-Wilk test). GraphPad Prism 9.0.0 was used to generate and assess the linear regressions. Pearson correlation coefficients were computed for normally distributed data, and nonparametric Spearman correlation was used for non-normally distributed data. Nonparametric Wilcoxon rank sum test was used for differential gene expression analysis. *p* < 0.05 was considered significant, and asterisks denote statistical significance level (*p < 0.05; **p < 0.01; ***p < 0.001; ****p < 0.0001).

## Results

### Effects of reduced *Ins2* gene dosage on hyperinsulinemia, obesity, and glucose homeostasis

We examined the effects of reduced *Ins2* gene dosage on PDAC development by crossing *Ptf1a*^*Cre*ER/WT^;*Kras*^LSL-G12D/WT^;*Ins1*^-/-^;*Ins2*^+/+^ mice with *Ptf1a*^WT/WT^;*Ins1*^-/-^;*Ins2*^+/-^ mice to activate mutant Kras expression in adult acinar cells and modulate insulin dosage [28, 36]. By this breeding scheme, we generated both *Ptf1a*^*Cre*ER^;*Kras*^LSL-G12D^;*Ins1*^-/-^;*Ins2*^+/+^ (PK-*Ins1*^-/-^;*Ins2*^+/+^) control mice and *Ptf1a*^*Cre*ER^;*Kras*^LSL-G12D^;*Ins1*^-/-^;*Ins2*^+/-^ (PK-*Ins1*^-/-^;*Ins2*^+/-^) experimental mice (Fig. 1A). Recombination and expression of the *Kras*^LSL-G12D^ allele was induced by injecting mice with tamoxifen at 4 weeks of age. To promote elevated insulin production and secretion (i.e. hyperinsulinemia), mice were fed a high-fat diet (HFD) after weaning (Fig. 1B).

In contrast to our previous study, which had more limited physiological data [19], body weight and fasting glucose levels were monitored every 4 weeks, and fasting insulin levels were measured every 3 months until euthanasia in both male and female mice (Fig. 1B). As expected from our previous studies using mice with reduced insulin gene dosage [24, 25, 37, 38], PK-*Ins1*^-/-^;*Ins2*^+/-^ mice had lower fasting insulin levels than PK-*Ins1*^-/-^;*Ins2*^+/+^ mice (Fig. 1C–D). This effect was observed continuously after 12 weeks of age for male mice (Fig. 1C) and was transiently present between 12 and 50 weeks of age for females (Fig. 1D), as previously reported [25]. Mice with reduced fasting insulin levels exhibited reduced weight gain in the context of HFD, without consistently affecting glucose homeostasis (Fig. 1E–H), consistent with our previous reports [22, 24, 25, 38]. When mice were 52 weeks old, glucose-stimulated insulin secretion tests, insulin tolerance tests, and glucose tolerance tests were conducted to examine glucose homeostasis more closely (Fig. 1I–N). At this age, PK-*Ins1*^-/-^;*Ins2*^+/+^ and PK-*Ins1*^-/-^;*Ins2*^+/-^ male mice secreted similar levels of insulin in response to intraperitoneal delivery of glucose; in females, we noted a clear trend in PK-*Ins1*^-/-^;*Ins2*^+/-^ mice towards reduced glucose-stimulated insulin secretion (Fig. 1I–J). There were no significant differences in insulin sensitivity between the PK-*Ins1*^-/-^;*Ins2*^+/+^ and PK-*Ins1*^-/-^;*Ins2*^+/-^ genotypes in either male or female mice despite males with reduced insulin production appearing slightly more insulin sensitive (Fig. 1K–L). Consistent with previous studies [24, 25], we found that male mice of both genotypes had weaker responses to insulin challenge than females (Fig. 1K–L). Glucose tolerance was generally similar between the genotypes, although male PK-*Ins1*^-/-^;*Ins2*^+/-^ mice were slightly, but significantly, more intolerant to glucose than *Ins2*^+/+^ littermate controls (Fig. 1M–N). We did not observe this effect in females (Fig. 1N). We also tested for potential differences in exocrine physiology by monitoring serum amylase levels [39], but found no differences between the genotypes (Fig. S1A-B).

In sum, the limited systemic physiological differences between the experimental PK-*Ins1*^-/-^;*Ins2*^+/-^ and control PK-*Ins1*^-/-^;*Ins2*^+/+^ mice offered an opportunity to examine the effects of reduced insulin production on PanIN formation in the absence of major changes in glucose homeostasis in both sexes.

### Effects of modestly reduced insulin on PanIN initiation

**Fig. 2:**
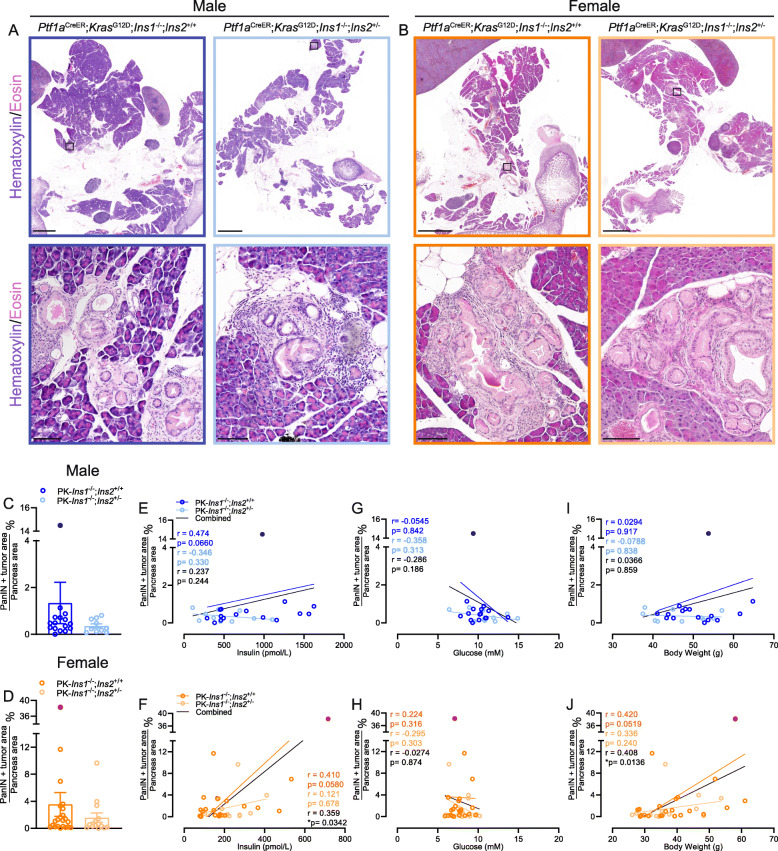
Effects of reduced hyperinsulinemia on pancreatic cancer initiation. **A**–**B** Representative whole section (top) and high-magnification (bottom) images of PK-*Ins1*^-/-^;*Ins2*^+/+^ and PK-*Ins1*^-/-^;*Ins2*^+/-^ male and female pancreata stained with hematoxylin and eosin. Scale bars: 2 mm (top) and 0.1 mm (bottom). **C**–**D** Quantification of percent of total pancreatic area occupied by PanINs and tumor in male (blue colors) and female (orange colors) PK-*Ins1*^-/-^;*Ins2*^+/+^ and PK-*Ins1*^-/-^;*Ins2*^+/-^ mice (*n* = 14–22) (dark blue and dark orange dots denote mice that developed PDAC). Correlations of composite PanINs plus tumor area with fasting insulin levels **E**–**F**, with fasting glucose levels **G**–**H**, or with body weight **I**–**J** in male and female PK-*Ins1*^-/-^;*Ins2*^+/+^ and PK-*Ins1*^-/-^;*Ins2*^+/-^ mice (*n* = 10-22)

Mice were euthanized at 57 weeks of age for histological analysis of the percent of total pancreatic area occupied by PanIN and tumor, or for scRNAseq analysis (see below). Similar to our previous study, we detected ductal lesions with histologic characteristics of low-grade PanIN (Fig. 2A–B), and only one male and one female mouse developed PDAC, and both of them were from the PK-*Ins1*^-/-^;*Ins2*^+/+^ genotype group. Similar to our previous study, the pancreatic area covered by PanIN and tumor in the PK-*Ins1*^-/-^;*Ins2*^+/+^ control mice was approximately twice that of the PK-*Ins1*^-/-^;*Ins2*^+/-^ experimental mice with reduced insulin levels (Fig. 2C–D, filled circles indicated mice with PDAC). In males, the pancreatic area covered by PanIN and tumor in PK-*Ins1*^-/-^;*Ins2*^+/+^ mice was 1.34% ± 0.88% compared with 0.36% ± 0.088% in the PK-*Ins1*^-/-^;*Ins2*^+/-^ mice. In females, control PK-*Ins1*^-/-^;*Ins2*^+/+^ mice had 3.54% ± 1.75% of the pancreas covered by PanIN and tumor compared with 1.58% ± 0.70% in the PK-*Ins1*^-/-^;*Ins2*^+/-^ mice. While not statistically significant, we observed a trend toward higher percent PanIN plus tumor area or PanIN area in females compared with males from the control genotype, with a similar trend occurring in the PK-*Ins1*^-/-^;*Ins2*^+/-^ genotype (Fig. S1C-E). While these results broadly support our previous findings in a mouse model that varied alleles of *Ins1* in an *Ins2*-null background [19]; there were far fewer PanIN lesions in both genotypes (~ 20% of pancreatic area in the previous study [19] vs < 4% in the current study for females), and the reduction in PanIN area was not statistically significant. The low lesion number in our current study may be related to the overall reduction in nonfat pancreatic area in the *Ins1*-null compared with the *Ins2*-null background (see below).

Next, we examined the correlations between PanIN plus tumor area and fasting insulin levels, glucose levels, and body weight in individual mice measured at 57 weeks of age, by pooled measurements (black) or within each group (colored) (Fig. 2E–J). We found that the relatively modest positive correlations between fasting insulin levels and PanIN plus tumor area were significant in female mice (Fig. 2F), but not in males (Fig. 2E). There was also a significant correlation between body weight and PanIN plus tumor area (Fig. 2J) in females, but not in males (Fig. 2I). There was no positive correlation between fasting glucose and PanIN plus tumor area in either sex (Fig. 2G–H), consistent with our previous findings [19]. Together, these data add support to our previous observations suggesting that hyperinsulinemia promotes PanIN development in females [19]. In males, the effects of hyperinsulinemia on PanIN development are less clear: despite a trend toward lower PanIN plus tumor area in the pancreata of males with reduced *Ins2* gene dose, PanIN plus tumor area did not correlate with insulin levels. While the reasons for this discrepancy between the sexes remains unclear, the relative insulin resistance we observed in both PK-*Ins1*^-/-^;*Ins2*^+/+^ and PK-*Ins1*^-/-^;*Ins2*^+/-^ males compared with females (Fig. 1 K–L) may provide one reason for the lack of correlation between circulating insulin and PanIN development in males.

### Acinar ductal metaplasia and adipocyte area in mice with reduced hyperinsulinemia

**Fig. 3:**
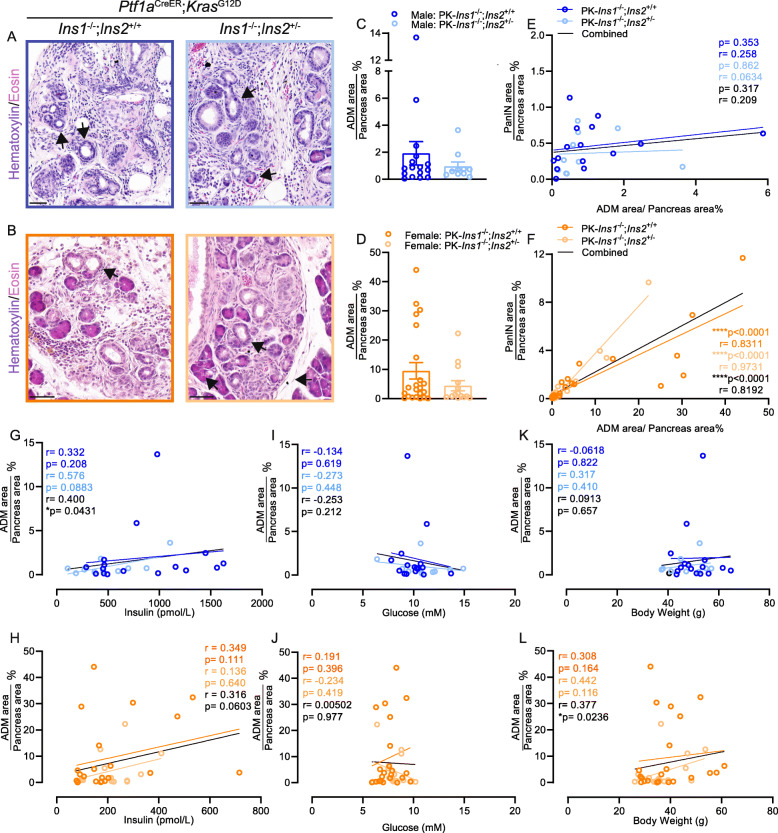
Altered ADM in mice with reduced hyperinsulinemia. **A**–**B** Representative high-magnification ADM (arrowheads) images of male (**A**) and female (**B**) PK-*Ins1*^-/-^;*Ins2*^+/+^ (left) and PK-Ins1^-/-^;Ins2^+/-^ (right) mouse pancreas sections stained with H&E. Scale bars: 0.05 mm. **C**–**D** Quantification of percent of total pancreatic area occupied by ADM in male and female mice of each genotype (*n* = 10–22). Correlations of composite ADM area with PanIN area in non-tumor bearing (**E–F**), fasting insulin levels (**G**–**H**), fasting glucose (**I**–**J**), or body weight (**K**–**L**) in male (**E**, **G**, **I**, **K**) and female (**F**, **H**, **J**, **L**) PK-*Ins1*^-/-^;*Ins2*^+/+^ and PK-*Ins1*^-/-^;*Ins2*^+/-^ mice (*n* = 10–16). Values are shown as mean ± SEM

Next, we measured the percent of total pancreatic area covered by ADM. ADM is histological evidence of normal acinar cells changing into ductal-like cells, and it can be induced by pancreatitis and during PanIN development [40]. We detected ADM in both male and female mice for each genotype (Fig. 3A–B). Similar to our PanIN area measurements, PK-*Ins1*^-/-^;*Ins2*^+/+^ mice had twice the amount of ADM area as PK-*Ins1*^-/-^;*Ins2*^+/-^ mice (1.92% ± 0.86% vs 0.96% ± 0.33%, respectively for males and 9.53% ± 2.84% vs 4.45% ± 1.74%, respectively for females), but the difference was not statistically significant (Fig. 3C–D). Also, consistent with the PanIN area, we found that the ADM area in PK-*Ins1*^-/-^;*Ins2*^+/+^ female mice was significantly higher than ADM area in PK-*Ins1*^-/-^;*Ins2*^+/+^ males (Fig. S1F). This trend was also present in the PK-*Ins1*^-/-^;*Ins2*^+/-^ genotype (Fig. S1G). When we next examined the correlations between percent ADM area and PanIN area, fasting insulin, fasting glucose, and body weight in individual mice at 57 weeks of age, for each genotype (orange or blue) or both together (black) (Fig. 3E–L), we found, as expected, that ADM area strongly correlated with PanIN area in non-tumor–bearing females of both genotypes (Fig. 3F). However, for male mice, there was no significant correlation between percent ADM area and PanIN only area (Fig. 3E). Despite a trend for the ADM area to correlate with fasting insulin in both sexes, only when the genotypes were combined did the ﻿models where percent ADM area correlates with fasting insulin in males (Fig. 3G) or body weight in females (Fig. 3L) significantly explain the spread of data points.

**Fig. 4:**
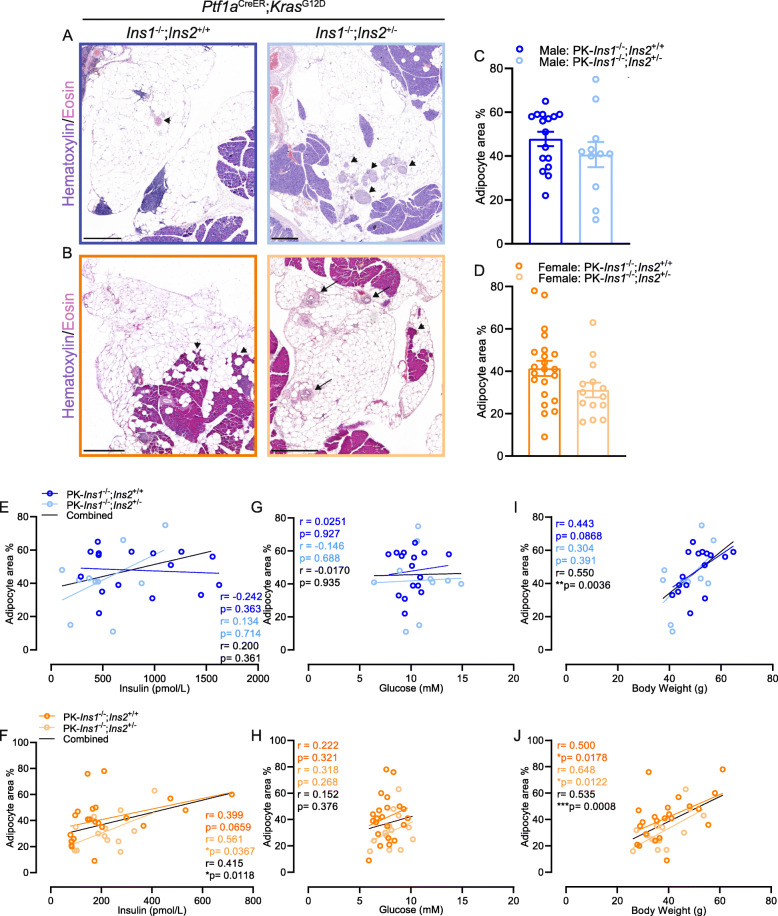
Altered pancreatic adipocyte area in mice with reduced hyperinsulinemia. **A**–**B** Representative high-magnification image of apparent adipocyte replacement of pancreas area in male (**A**) and female (**B**) PK-*Ins1*^-/-^;*Ins2*^+/+^ (left) and PK-*Ins1*^-/-^;*Ins2*^+/-^ (right) mice stained with H&E. Black arrows point to residual ducts. Arrowheads point to remaining islets, acinar cells, and blood vessel. Scale bars: 0.5 mm. **C**–**D** Quantification of percent of total pancreatic area occupied by adipocytes in male and female mice of each genotype (*n*= 11–22). Correlations of adipocyte area with fasting insulin levels (**E**–**F**), fasting glucose levels (**G**–**H**), or body weight (**I**–**J**) for male (**E**, **G**, **I**) and female (**F**, **H**, **J**) PK-*Ins1*^-/-^;*Ins2*^+/+^ (dark colors) and PK-*Ins1*^-/-^;*Ins2*^+/-^ (light colors) mice (*n* = 11–22). Values are shown as mean ± SEM

One surprising observation from our histological analyses was the significant amount of pancreatic area that had been replaced by adipocytes in our PK-*Ins1*^-/-^ mouse model. This is not a phenomenon that we had previously observed in our PK-*Ins2*^-/-^ model [19]. As the representative images show (Fig. 4A–B), we often observed pancreatic lobules with few residual normal acinar, ductal, or endocrine cells left among large numbers of adipocytes. The percent of pancreatic area replaced by adipocytes was not significantly different between PK-*Ins1*^-/-^;*Ins2*^+/+^ and PK-*Ins1*^-/-^;*Ins2*^+/-^ mice (Fig. 4C–D), suggesting this phenotype was specifically associated with loss of *Ins1* gene. Male mice had a slightly higher percent adipocyte area than female mice for both genotypes; overall about 30–50% of the pancreatic area was occupied by adipocytes. The fatty replacement affected the overall parenchymal area, as we found compared with PK-*Ins2*^-/-^ female mice; PK-*Ins1*^-/-^ female mice had significantly less pancreatic area (PK-*Ins2*^-/-^ male mice did not reach a comparable age and were not assessed) (Fig. S1H). It is possible that this fatty replacement could have affected the overall number of PanIN lesions, because of a relative lack of Ptf1a-positive acinar cells. Interestingly, there was a significant correlation between the percent of adipocyte area and fasting insulin for female, but not male mice (Fig. 4E–F). We observed no correlation between percent of adipocyte area and fasting glucose levels for either sex (Fig. 4G–H). However, as expected [25], the percent adipocyte area did correlate with body weight in both sexes (Fig. 4I–J). The underlying cause of fatty replacement in the PK-*Ins1*^-/-^ mice is unknown and could be multifactorial, but the forces driving fat accumulation could potentially influence the accumulation of PanIN lesions, especially in females. This is supported by the significant and strong correlation between the percent of pancreatic area occupied by PanIN vs adipocytes in non-tumor–bearing in female, but not male, mice (Fig. S1I-J). We have previously observed this fatty replacement of normal parenchyma in another colony of *Ins1*^-/-^ mice (unpublished observations); therefore, we do not believe this phenomenon is specific to the exposure of mice to tamoxifen or the influence solely of the PK mutant alleles.

### Single-cell transcriptomics reveals effects of hyperinsulinemia on cell type–specific gene expression

**Fig. 5:**
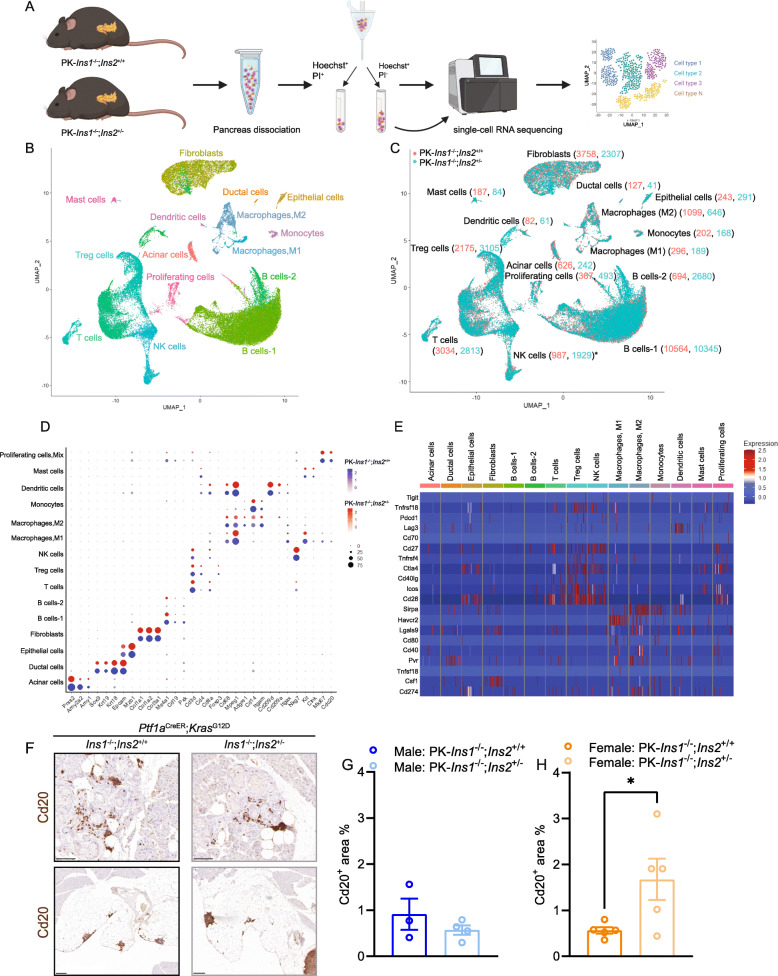
scRNAseq analysis reveals effects of hyperinsulinemia on cell type-specific gene expression. **A** Schematic describing the single-cell transcriptomics experimental design and analysis. Six mouse pancreata from each genotype were dissociated to single cells, and the samples were sorted for hoechst^+^ and PI^-^ live cells. The live single cells were sequenced and clustered in uniform manifold approximation and projection (UMAP) space. **B** Unsupervised clustering of cells from 6 PK-*Ins1*^-/-^;*Ins2*^+/+^ and 6 PK-*Ins1*^-/-^;*Ins2*^+/-^ mice pancreata, represented as an UMAP plot. **C** Numbers of cells from PK-*Ins1*^-/-^;*Ins2*^+/+^ (orange) and PK-*Ins1*^-/-^;*Ins2*^+/-^ (green) mice for each cell type. The asterisk indicates a significant difference in the number of NK cells between the genotypes. **D** Dot plot showing selected cell type-specific markers for identifying the cell type for each cluster. The size of dots represents the fraction of cells expressing the markers. The PK-*Ins1*^-/-^;*Ins2*^+/+^ mouse data are shown in blue and PK-*Ins1*^-/-^;*Ins2*^+/-^ mouse data are shown in red. The intensity of color indicates the average expression of marker genes for each cell type. **E** Single-cell expression of immune checkpoint receptors and ligands in each identified cell population in both genotypes. **F** Immunohistochemistry of Cd20 (B cell marker) for PK-*Ins1*^-/-^;*Ins2*^+/+^ and PK-*Ins1*^-/-^;*Ins2*^+/-^ pancreata. Scale bars: 0.1 mm (up) and 0.2 mm (down). **G**–**H** The quantification of Cd20^+^ area for PK-*Ins1*^-/-^;*Ins2*^+/+^ and PK-*Ins1*^-/-^;*Ins2*^+/-^ male (**G**) and female (**H**) mice

To investigate the molecular effects of hyperinsulinemia in the context of PDAC initiation in an unbiased and cell type–specific manner, we undertook scRNAseq. At 57 weeks of age, we collected pancreata from 6 PK-*Ins1*^-/-^;*Ins2*^+/+^ control mice and 6 PK-*Ins1*^-/-^;*Ins2*^+/-^ experimental mice (all fasted for 4 h), dispersed them into single cells, and FACS purified live cells for single-cell RNA sequencing (Fig. 5A). We performed scRNAseq analysis separately for each animal. In total, 49,835 single cells passed quality control tests and were clustered into 15 clusters (Fig. 5B–C). These cell clusters were assigned cellular identities based on the expression of known markers, which were commonly used by other studies [31–33] (Fig. 5D). We were able to identify acinar cells, ductal cells, and fibroblasts. The majority of cells that survived dispersion and passed transcriptomics quality controls were immune cells including: T cells, T regulatory cells (Treg), B cells, natural killer (NK) cells, macrophages (both M1 and M2 macrophages), monocytes, dendritic cells, and mast cells (Fig. 5B–D, and Fig. S2D). We also classified a separate cluster of proliferating cells marked by high expression of *Mki67*. This proliferating cell cluster included multiple immune cell types, such as T cells, B cells, and NK cells, as well as epithelial cells (Fig. S2A-C). Because there was clearly inconsistent detection of cell populations across genotypes and sex, we chose to combine the sexes before performing comparisons between genotypes. There were no significant differences in the numbers of cells per cluster between the genotypes, with the exception of there being twice as many NK cells in mice with reduced insulin (Fig. 5C). Analysis of cell type–specific markers showed that cell identities were generally comparable between genotypes (Fig. 5D). Consistent with previous human PDAC single transcriptomics analysis [31], we were able to identify 3 different fibroblast subtypes within the fibroblast cell cluster from pancreata undergoing metaplastic changes, including inflammatory fibroblasts, myofibroblastic fibroblasts, and antigen-presenting fibroblasts (Fig. S2E). Consistent with the findings of Steele *et al.* [32], the PanIN microenvironment in our model displayed evidence of immunosuppression. Specifically, we observed T cells, Treg cells, and NK cells expressed immune checkpoint receptors like *Cd28*, *Ctla4*, *Icos*, *Tnfrsf18*, and *Cd27,* while macrophages expressed checkpoint ligands, including *Sirpa*, *Havcr2*, *Pvr*, and *Lgals9* (Fig. 5E). However, there was no significant difference in expression of these immune checkpoint receptors and ligands between the two genotypes (see differential gene expression presented below, Table S2). This suggested even at the PanIN formation stage, the three types of fibroblasts and the immunosuppressive microenvironment were present, but unaffected by insulin gene dosage. Our scRNAseq analysis was rich with B cells (Fig. 5B–C). To confirm the presence of B cells in our mouse pancreata, we performed IHC staining for B cell marker, Cd20 (encoded by *Ms4a1*). We observed B cells surrounding PanINs and, interestingly, we also observed aggregates of B cells associated with the fatty replacement of the parenchyma (Fig. 5F). We did not observe a significant difference in Cd20^+^ area between the genotypes for male mice (Fig. 5G); however, there was a significant increase of Cd20^+^ area in female PK-*Ins1*^-/-^;*Ins2*^+/-^ mice compared with female PK-*Ins1*^-/-^;*Ins2*^+/+^ mice (Fig. 5H). This was consistent with our scRNAseq analysis that also showed there were more cells present in the B cluster in female PK-*Ins1*^-/-^;*Ins2*^+/-^ compared with female PK-*Ins1*^-/-^;*Ins2*^+/+^ mice. Using IHC staining, we also confirmed the presence of other immune cells, including macrophages, Treg cells, and Cd8^+^ T cells in the PanIN microenvironment (Fig. S3A-C). There were a large number of macrophages present surrounding ADM and PanIN lesions, but we did not find a significant difference in the infiltration of macrophages around each type of lesion between the genotypes, and only a relatively small number of Foxp3^+^ and Cd8^+^ T cells were observed regardless of genotype (Fig. S3A-D). Overall, our IHC staining supported our single-cell transcriptomic analysis and confirmed the presence of multiple immune cell types in the neoplastic microenvironment of our mouse model.

**Fig. 6:**
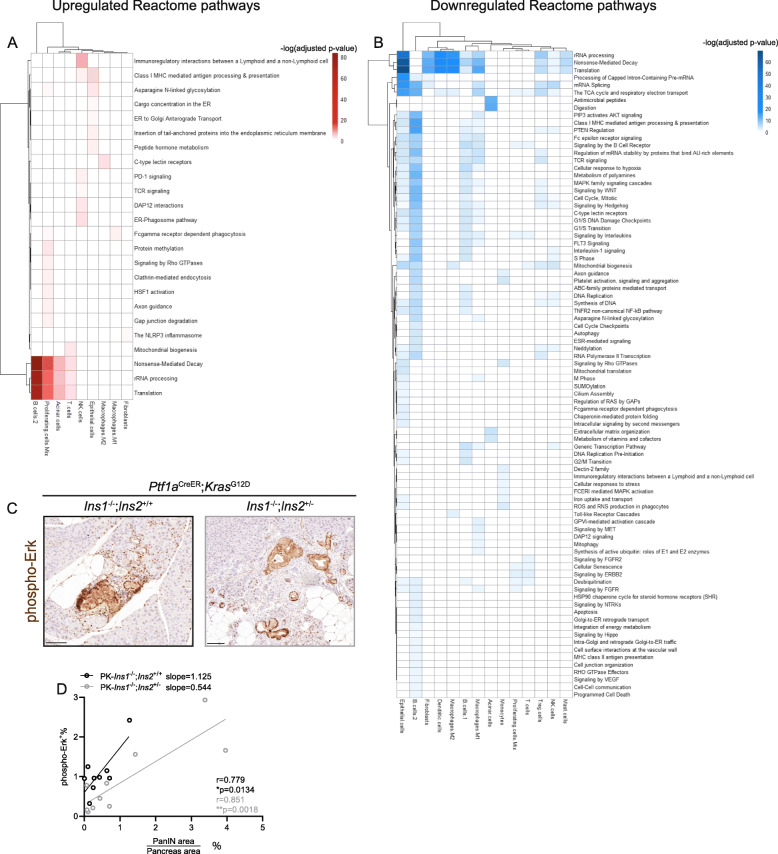
scRNAseq analysis reveals the pathways that are altered in each cell type by hyperinsulinemia. **A** Heatmap showing Reactome pathways that are upregulated in PK-*Ins1*^-/-^;*Ins2*^+/-^ mice when compared with PK-*Ins1*^-/-^;*Ins2*^+/+^ mice for each cell type. The intensity of color indicates the negative log_10_ of adjusted *p* value. **B** Heatmap of Reactome pathways that are downregulated in PK-*Ins1*^-/-^;*Ins2*^+/-^ mice when compared with PK-*Ins1*^-/-^;*Ins2*^+/+^ mice for each cell type. The intensity of color indicates the negative log_10_ of adjusted *p* value. **C** Immunohistochemistry of phospho-Erk for PK-*Ins1*^-/-^;*Ins2*^+/+^ and PK-*Ins1*^-/-^;*Ins2*^+/-^ pancreata. Scale bars: 0.1 mm. **D** The correlation of composite PanINs area with phospho-Erk positive area. *p < 0.05, **p < 0.01

After confirming the cell identities, we generated a list of genes that were differentially expressed between genotypes for each cell type (Table S2). To have a more comprehensive understanding of the function of these differentially expressed genes, we performed pathway enrichment analysis using Reactome and to identify the pathways that were up- (Fig. 6A) and downregulated (Fig. 6B) in PK-*Ins1*^-/-^;*Ins2*^+/-^ experimental mice compared with PK-*Ins1*^-/-^;*Ins2*^+/+^ control mice. B cells-1, B cells-2, epithelial cells, and M1 macrophages were the cell types that had the most altered pathways (Fig. 6A–B). Specifically, the pathways that were most significantly altered were rRNA processing, nonsense-meditated decay, and translation, and they were also the pathways that were consistently altered across multiple cell types (Fig. 6A–B). Interestingly, these pathways were downregulated in epithelial cells, fibroblasts, dendritic cells, macrophages, B cells-1, Treg, NK cells, and mast cells of mice with reduced insulin. However, they were upregulated in B cells-2, proliferating cells, and acinar cells of mice with reduced insulin (Fig. 6A–B). We also found pathways that were only altered in acinar cells, which initiate the metaplasia in these mice. For instance, antimicrobial peptides and digestion enzymes were downregulated in acinar cells from PK-*Ins1*^-/-^;*Ins2*^+/-^ experimental mice (Fig. 6B). The antimicrobial peptide category highlighted that the *Reg3a*, *Reg3b*, *Reg3d*, and *Reg3g* genes, which are known to be induced by inflammation and may have antimicrobial roles [41, 42], were significantly downregulated in acinar cells from mice with reduced insulin production (Fig. S2F). This suggests that inflammation surrounding acinar cells in the histologically normal pancreatic areas due to HFD treatment [43, 44] might be reduced in mice with less insulin. Reg proteins are reported to promote pancreatic tumorigenesis [45–47] and future studies may identify a role for these proteins in mediating the effects of hyperinsulinemia in promoting inflammation and conversion of acinar cells into duct-like cells.

Somewhat expectedly, pathways involved in insulin signaling, like “PIP3 activates AKT signaling”, “PTEN regulation”, and “MAPK family signaling cascades”, were downregulated in mice with reduced insulin production. They were downregulated in several cell types, but most clearly in B cells, epithelial cells, and M1 macrophages (Fig. 6B). There was also a downregulation of genes involved in cell cycle pathways in epithelial cells, B cells, macrophages, Treg cells, and NK cells from mice with reduced insulin production (Fig. 6B). To validate the pathway enrichment analysis, we performed IHC staining of phospho-Erk. As expected, the PanIN cells had dark staining indicating robust phospho-Erk signaling. We also observed some non-epithelial cell types and acinar cells had high levels of phospho-Erk (Fig. 6C). To see whether the latter areas contributed significantly to the overall phospho-Erk activity in the pancreas, we performed correlation analyses with the PanIN area per individual. As expected, there was a significant correlation between the phospho-Erk^+^ area and PanIN plus tumor area for both genotypes (Fig. 6C–D). However, the slope of PK-*Ins1*^-/-^;*Ins2*^+/+^ mice was larger than the slope of PK-*Ins1*^-/-^;*Ins2*^+/-^ mice, which suggested there were more non-PanIN cells with phospho-Erk positivity in PK-*Ins1*^-/-^;*Ins2*^+/+^ mice compared with PK-*Ins1*^-/-^;*Ins2*^+/-^ mice (Fig. 6D). This was consistent with our pathway enrichment analysis that MAPK-ERK signaling pathway was downregulated in epithelial and non-epithelial cell types of PK-*Ins1*^-/-^;*Ins2*^+/-^ mice. Altogether, the scRNAseq analysis demonstrated the transcriptomics of epithelial cells and immune cells were significantly altered by hyperinsulinemia. This suggests that hyperinsulinemia might directly and indirectly affect PanIN development through regulating the epithelial and immune cells, respectively, in the PanIN microenvironment.

## Discussion

The goal of this study was to investigate the effects of reduced *Ins2* gene dosage on HFD-induced hyperinsulinemia, PanIN initiation, and cell type–specific gene expression in the context of acinar cell–specific expression of mutant Kras. The results of the present study extend our previous findings [19], which implicate hyperinsulinemia as a causal factor in pancreatic cancer initiation, and provide the first molecular insights into the cell-specific mechanisms involved.

Despite the strong epidemiological link between hyperinsulinemia and pancreatic cancer, the specific reduction of insulin is required to formally test the hypothesis that insulin plays a causal role. Our previous study was the first to demonstrate that endogenous hyperinsulinemia contributes to cancer development, primarily the earliest events, using mice with reduced dosage of *Ins1* in a *Ins2*-null genetic background [19]. Unfortunately, in that study, male PK-*Ins1*^+/-^;*Ins2*^-/-^ mice developed hyperglycemia at a very young age because of insufficient endogenous insulin production, which limited our conclusions to female mice [19]. In the present investigation, we were eager to extend our observations to both sexes and indeed, we found that male PK-*Ins1*^-/-^;*Ins2*^+/-^ mice were able to maintain glucose homeostasis and be studied long-term. This is consistent with previous studies showing that limiting *Ins2* gene dosage prevented hyperinsulinemia without pronounced effects on glucose homeostasis [24]. Our data show that reduced *Ins2* gene dosage led to a moderate reduction in fasting insulin levels without broad effects on glucose homeostasis, in both male and female mice. It should be noted that circulating insulin levels in female mice, even with both *Ins2* alleles, are only ~ 25% of that seen in male mice. We also noted in female mice that insulin levels were not different at 1 year of age between genotypes, mirroring the transient compensation we have previously observed in *Ins1*-null model [25, 38]. Collectively, these observations illustrate that a reduction in *Ins2* gene dosage results in a relatively mild manipulation of circulating insulin in the first year of life. Because *Ins2* is the ancestral gene and contributes to ~ 2/3 of secreted insulin [21, 23], fasting insulin levels were still relatively high for our PK-*Ins1*^*-/-*^ mouse model compared with the previously studied PK-*Ins2*^*-/-*^ mouse model (Fig. 1C–D vs [19]).

Consistent with the previous study [19], in this study, only 2 animals developed PDAC; therefore, we focused on quantifying the effects of reduced insulin on PanIN and ADM development, as well as the observed fatty replacement. Interestingly, in the present study, only about 1–4% pancreas was occupied by PanIN lesions for our PK-*Ins1*^*-/-*^ mouse model, compared with the 15–30% of pancreas that was occupied by PanIN lesions for our previous PK-*Ins2*^*-/-*^ mouse model [19]. Another major histological difference between this study and our previous one was the observation of a significant amount of fatty replacement of the parenchyma in our PK-*Ins1*^-/-^ mouse model. Approximately 30–50% of the pancreas was replaced by adipocytes. Unfortunately, because we did not collect pancreata at earlier time points and the 10x genomics scRNAseq platform does not efficiently capture adipocytes, our study could not address when the fatty replacement occurred or the potential transcriptional programs altered in adipocytes, which could provide us some insight into the cause of fatty replacement. The presence of fat in the parenchyma was previously observed, but not reported, in our previous *Kras*^WT^;*Ins1*^-/-^;*Ins2*^+/+^ mouse studies (unpublished observations from animal cohorts in [24, 25, 38]). This suggests the *Ins1*^-/-^ genetic background may contribute to replacement of parenchyma with fat and the relative lack of ductal metaplasia; however, future studies are needed to examine this hypothesis. In addition, pancreatitis can also induce acinar cell necrosis or apoptosis, which is subsequently replaced by adipocytes [48, 49]. HFD-induced obesity can also cause fat accumulation in the exocrine parenchyma [48, 50], but we did not observe this extent of fat accumulation with the same diet in our previous mouse model [19]. Together it seems that the combined effects of Kras-associated inflammation, HFD, and the *Ins1*^-/-^ genetic background may have resulted in fat displacing ~ 2/3 of normal pancreatic parenchyma in our PK-*Ins1*^*-/-*^ mouse model. Therefore, the loss of acinar cells may explain why fewer and more variable numbers of PanIN lesions developed in our PK-*Ins1*^*-/-*^ mouse model. Nevertheless, circulating insulin still significantly correlated with PanIN plus tumor area in female mice, confirming our previous report with another insulin gene dosage configuration. Our histopathological analysis also showed that PK-*Ins1*^-/-^;*Ins2*^+/-^ mice had a ~ 50% reduction in percent PanIN area compared with PK-*Ins1*^-/-^;*Ins2*^+/+^ mice, which is similar to our previous findings. Although the pairwise comparison between genotypes did not reach statistical significance in the present study [19], our findings still support a role for hyperinsulinemia promoting PanIN initiation from acinar cells sustaining mutations in the oncogene *Kras*.

Although we did not longitudinally track the dynamic levels of insulin across time, we performed analysis of glucose-stimulated insulin secretion and noted that response to glucose challenge was not significantly different between the PK-*Ins1*^-/-^;*Ins2*^+/+^ and PK-*Ins1*^-/-^;*Ins2*^+/-^ genotypes of either sex. However, the change in insulin appeared to differ between female PK-*Ins1*^-/-^;*Ins2*^+/-^ compared with PK-*Ins1*^-/-^;*Ins2*^+/+^ controls. While this genotype effect on glucose-stimulated insulin secretion between the sexes was consistent with previous findings [24, 25], our current analysis was limited to one time point. Another limitation of our study is that we were not able to rule out contributions of indirect effects of obesity on PanIN development. Other studies have suggested that obesity can promote PanIN development through sustained inflammation, dysregulated metabolism, or aberrant islet β-cell expression of peptide hormone cholecystokinin (Cck) [43, 51–53]. Interestingly, the induction of Cck, a stimulant of acinar cell secretion, in aberrant islets in the leptin-deficient–induced obesity model studied by Chuang *et al*. suggests communication between the islet and acinar cell compartment occurs [51]. To examine whether insulin similarly communicates with acinar cells in the local pancreatic neighborhood, future studies will inhibit insulin receptor signaling in the *Kras*^G12D^-expressing acinar cells to determine the direct effects of hyperinsulinemia on PanIN development. Additionally, studies to test the role of obesity in the absence of hyperinsulinemia are necessary to further address the role of each factor in PanIN development.

Pancreatic cancer is one of the most stroma-rich solid tumor types, and immune cells in the microenvironment play important roles at both early and late stages of PDAC development [54–57]. In our single-cell transcriptomics analysis, the primary cell types analyzed were immune cells, including T cells, B cells, macrophages, NK cells, and dendritic cells. We observed a significant change in the number of NK cells present in our scRNA analyses and a trend towards less macrophages by F4/80 IHC, which could suggest that there are changes in the immune microenvironment during PanIN formation in mice with reduced insulin. However, consistent with previous analyses of the immune cells in human PDAC or mouse models of PDAC, immune cells from our samples also expressed immune checkpoint receptors regardless of genotype suggesting that the suppressed immune microenvironment is not affected by the reduction of insulin. However, the major signaling pathways downstream of insulin (e.g., MAPK-ERK, PI3K-AKT, cell cycle, and translation pathways) were downregulated in immune cells from mice with reduced insulin production. MAPK-ERK and PI3K-AKT signaling pathways and their downstream signaling cascades are well-established regulators of multiple immune cell types [12, 58, 59]. Therefore, there may still be differences in immune cell activities that were not captured by our transcriptome analyses. Future studies should examine the proteome and phospho-proteome of these cells.

The largest immune cell population identified by our single cell RNAseq was B cells. Different from other immune cell types, B cells are not well studied in the context of PDAC development, especially at PanIN initiation stage. B cell infiltration around PanIN and PDAC was previously observed in a murine PDAC model and human samples [32, 60, 61]. By IHC, we found that B cells are located, as expected, in the lymph nodes, around metaplastic areas, as well as in the areas of fatty infiltration. The large numbers of B cells in our dataset may have arisen as a consequence of selective enrichment of the pancreatic lymph nodes during cellular dissociation. However, the pronounced B cell infiltration at the fatty replacement regions suggests B cells might be associated with the fatty replacement process, although this remains to be tested directly. Regardless of their origin, the B cells in our analyses had the greatest number of pathways altered significantly by hyperinsulinemia. Our staining demonstrated that female mice with reduced insulin production had significantly more B cells than control mice, and this was consistent with the trend observed in our scRNA-seq analysis. The role of B cells in PDAC appears to be complex according to the few studies in which it has been investigated. Some studies demonstrated that B cells secreted interleukin-35 and promoted tumor progression, while Spear et al. showed that B cells were proinflammatory and limited PDAC development, but these genes were not expressed by B cells in our scRNA-seq analysis of early pancreatic changes [61–63]. Future studies are therefore required to better understand how hyperinsulinemia affects B cells and how B cells contribute to PanIN development under these conditions. Overall, our scRNAseq analysis suggested hyperinsulinemia might contribute to PanIN development through changes in the immune microenvironment.

A limitation of our single-cell transcriptomics was that relatively few acinar cells survived the pancreas dispersion and were captured in the "live" gate by FACS . This was somewhat expected given the fragility of acinar cells and meant that we had less cell-level power to detect differences in gene expression and that we may not have been assessing gene expression in an exceptionally robust sub-grouping of acinar cells. Nevertheless, within the limited data, we observed a significant downregulation of *Reg3a*, *Reg3b*, *Reg3d*, and *Reg3g* in acinar cells from PK-*Ins1*^-/-^;*Ins2*^+/-^ mice. Reg proteins have been shown to promote pancreatic carcinogenesis, especially inflammation-linked pancreatic carcinogenesis [45–47]. Inflammation in pancreas can cause ADM and accelerate PanIN progression [36, 64] and therefore, the decrease of *Reg* transcripts in PK-*Ins1*^-/-^;*Ins2*^+/-^ mice is consistent with the reduction of PanINs and inflammation in those mice. Future studies may seek to directly manipulate Reg proteins in the context of hyperinsulinemia and pancreatic cancer.

One unexpected finding from our study was the observation that males and females showed differences in the way that sex affects the induction of metaplasia and neoplasia. For example, the percentage of pancreas replaced by ADM was higher in PK-*Ins1*^-/-^;*Ins2*^+/+^ females than in males (Fig. S1F), with a similar trend in the PK-*Ins1*^-/-^;*Ins2*^+/-^ genotype (Fig S1G). There was also a sex difference in the correlation between PanIN and ADM area (Fig. 3E–F), where PanIN area showed a significant positive correlation with ADM area in females, but not males, in animals without tumors. While it remains unclear whether the differences we observe between the sexes are due to methodological (e.g., tamoxifen pharmacology) or biological factors (e.g., frequency of tumor initiation), our findings suggest that future studies should examine and analyze males and females separately in the Ins1^-/-^ genetic background and potentially other genetic backgrounds. This will allow a more comprehensive examination of whether known sex differences in the regulation and physiological action of insulin in the body contribute to tumor development [65–69].

In future studies, it will be important to characterize the specific downstream changes for each immune cell type at the protein level. It will also be important to manipulate insulin signaling components, including the insulin receptor, in acinar cells, immune cells, and other components of the PanIN microenvironment to determine which cells are predominately being affected by changes in insulin. Our study represents an important first step in understanding the molecular effects of hyperinsulinemia on all the cell types present in the context of early-stage pancreatic cancer.

## Conclusions

The present study showed that mice with reduced hyperinsulinemia trended to have less pancreatic area covered by PanIN and ADM, consistent with our previous data demonstrating that hyperinsulinemia can contribute causally to PanIN development. The scRNA-seq analysis demonstrated that hyperinsulinemia affected the immune cell composition in the PanIN microenvironment and altered cellular pathways involved in or targets of insulin signaling, such as the MAPK-ERK pathways and protein translation. Our study represents an important first step in understanding the molecular effects of hyperinsulinemia on all the cell types present during initiation of pancreatic cancer.

## Supplementary information

**Additional file 1:** **Table S1.** Primary and secondary antibodies.

**Additional file 2:** **Table S2.** Genes differentially expressed between genotypes for each cell cluster.

**Additional file 3: Fig. S1.** Amylase activity and pancreatic area of PK-*Ins1*^-/-^;*Ins2*^+/+^ and PK-*Ins1*^-/-^;*Ins2*^+/-^ mice and the sex difference in PanIN development. **A-B** The amylase activity in male **(A)** and female mice **(B)** for each genotype. **C-D** The comparison of percent of total pancreatic area occupied by PanINs and tumor **(C)** or only PanINs (excluding tumor bearing mice) **(D)** in male and female PK-*Ins1*^-/-^; *Ins2*^+/+^ mice (*n*= 15-22) (dark blue and dark orange dots denote mice that developed tumors). **E** The comparison of percent of total pancreatic area occupied by only PanINs in male and female PK-*Ins1*^-/-^; *Ins2*^+/-^ mice (*n*= 10-14). **F** The comparison of percent of total pancreatic area occupied by ADM in male and female PK-*Ins1*^-/-^; *Ins2*^+/+^ mice (*n*= 16-22). **G** The comparison of percent of total pancreatic area occupied by ADM in male and female PK-*Ins1*^-/-^; *Ins2*^+/-^ mice (*n*= 10-14). **H** The total pancreatic area for mice in an *Ins2*-null background or in an *Ins1*-null background. **I-J** Correlations of adipocyte area with PanIN area in non-tumor bearing male **(I)** and female **(J)** PK-*Ins1*^-/-^;*Ins2*^+/+^ (dark colors) and PK-*Ins1*^-/-^;*Ins2*^+/-^ (light colors) mice (*n* = 10-22). ****p<0.0001. Values are shown as mean ± SEM.

**Additional file 4: Fig. S2.** scRNAseq analysis shows there are multiple cell types in the proliferating cell cluster and there are 3 types of fibroblasts. **A** Unsupervised sub-clustering of the cluster containing proliferating cells, represented as an UMAP plot. The proliferating cell cluster contains proliferating T cells, B cells, Naïve B cells, NK cells and epithelial cells. **B** Numbers of cells from PK-*Ins1*^-/-^;*Ins2*^+/+^ (orange) and PK-*Ins1*^-/-^;*Ins2*^+/-^ (green) mice for each cell type. **C** Violin plot showing the expression level of selected cell type-specific markers for identified cell types within the proliferating cell cluster. **D** Expression level of the typical markers for identifying M1 macrophages and M2 macrophages for each genotype. **E** Violin plots showing the expression level of selected markers for inflammatory, myofibroblastic and antigen-presenting fibroblasts. **F** The differential expression of *Reg3a*, *Reg3b*, *Reg3d*, and *Reg3g* genes in acinar cells between PK-*Ins1*^-/-^;*Ins2*^+/+^ and PK-*Ins1*^-/-^;*Ins2*^+/-^ mice.

**Additional file 5: Fig. S3.** Multiple immune cells present around PanIN and ADM lesions. **A-C** Immunohistochemistry of F4/80 **(A)**, Foxp3 **(B)**, and Cd8 **(C)** for PK-*Ins1*^-/-^;*Ins2*^+/+^ and PK-*Ins1*^-/-^;*Ins2*^+/-^ pancreata. (Representative Cd8^+^ T cells in lymph nodes, **C** inset). **D** Quantification of F4/80 positive area per lesion area for each ADM, PanIN and tumor area. No PK-*Ins1*^-/-^;*Ins2*^+/-^ mice developed PDAC. (**A**: Scale bars: 0.1mm. **B-C:** Scale bars: 0.05mm).

## Data Availability

All data generated and analyzed in this study is included within the article and additional files or is available from the corresponding author on request.

## Abbreviations

PanIN: Pancreatic intraepithelial neoplasia
ADM: Acinar-to-ductal metaplasia
PDAC: Pancreatic ductal adenocarcinoma
HFD: High-fat diet
H&E: Hematoxylin and eosin
IHC: Immunohistochemical
scRNAseq: Single-cell RNA sequencing
UMAP: Uniform manifold approximation and projection
SEM: Standard error of the mean
Treg: T regulatory cells
NK: Natural killer cells
Cck: Cholecystokinin
