# Supplementary material for: Effects of hyperinsulinemia on pancreatic cancer development and the immune microenvironment revealed through single-cell transcriptomics
Source: Cancer Metab. 2022 Feb 21;10:5. doi: 10.1186/s40170-022-00282-z (PMC8862319; doi:10.1186/s40170-022-00282-z)
Supplement: Supplementary file 1 — Additional file 1: Table S1. Primary and secondary antibodies. [file 40170_2022_282_MOESM1_ESM.docx]

**Primary Antibodies**

| Antigen | Source | Catalog | Dilution | Species | Antigen retrieval |
| --- | --- | --- | --- | --- | --- |
| Cd20 | Cell Signalling Technology | 70168T | 1:500. | Rabbit | Low pH buffer 200mL 10mM citrate buffer at pH 6 |
| F4/80 | Invitrogen eBioscience Thermo Fisher | 14-4801-82 | 1:100. | Rat | No antigen retrivel was performed |
| Foxp3 | Invitrogen | 14-5773-82 | 1:200. | Rat | Low pH buffer 200mL 10mM citrate buffer at pH 6 |
| Cd8 | Invitrogen eBioscience Thermo Fisher | 14-0808-82 | 1:1000. | Rat | Low pH buffer 200mL 10mM citrate buffer at pH 6 |
| p-Erk | Cell Signalling Technology | 4370S | 1:500. | Rabbit | Low pH buffer 200mL 10mM citrate buffer at pH 6 |

**Secondary Antibodies**

| Antigen | Source | Catalog | Dilution | Species |
| --- | --- | --- | --- | --- |
| Rat | Vector Laboratories | MP-7404 | 1:2. | Goat |
| Rabbit | Jackson ImmunoResearch laboratories, Inc. | 711-065-152 | 1:500. | Donkey |
| Rat | Jackson ImmunoResearch laboratories, Inc. | 712-065-150 | 1:500. | Donkey |
