# Supplementary figures and images for: Effects of hyperinsulinemia on pancreatic cancer development and the immune microenvironment revealed through single-cell transcriptomics
Source: Cancer Metab. 2022 Feb 21;10:5. doi: 10.1186/s40170-022-00282-z (PMC8862319; doi:10.1186/s40170-022-00282-z)

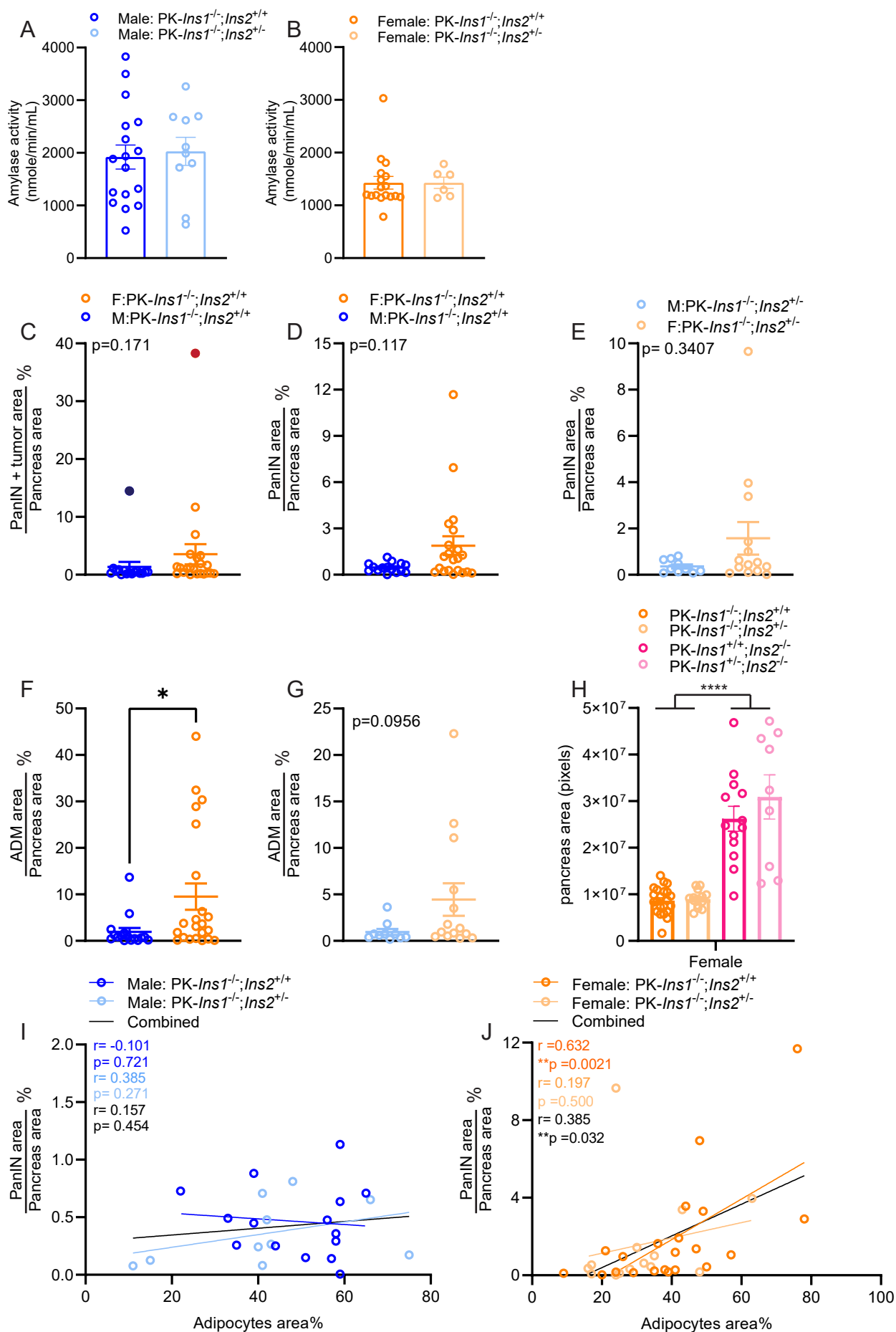

Supplemental Figure 1

Supplement: Supplementary file 3 — Additional file 3: Fig. S1. Amylase activity and pancreatic area of PK-Ins1-/-;Ins2+/+ and PK-Ins1-/-;Ins2+/- mice and the sex difference in PanIN development. A-B The amylase activity in male (A) and female mice (B) for each genotype. C-D The comparison of percent of total pancreatic area occupied by PanINs and tumor (C) or only PanINs (excluding tumor bearing mice) (D) in male and female PK-Ins1-/-; Ins2+/+ mice (n= 15-22) (dark blue and dark orange dots denote mice that developed tumors). E The comparison of percent of total pancreatic area occupied by only PanINs in male and female PK-Ins1-/-; Ins2+/- mice (n= 10-14). F The comparison of percent of total pancreatic area occupied by ADM in male and female PK-Ins1-/-; Ins2+/+ mice (n= 16-22). G The comparison of percent of total pancreatic area occupied by ADM in male and female PK-Ins1-/-; Ins2+/- mice (n= 10-14). H The total pancreatic area for mice in an Ins2-null background or in an Ins1-null background. I-J Correlations of adipocyte area with PanIN area in non-tumor bearing male (I) and female (J) PK-Ins1-/-;Ins2+/+ (dark colors) and PK-Ins1-/-;Ins2+/- (light colors) mice (n = 10-22). ****p<0.0001. Values are shown as mean ± SEM. [file 40170_2022_282_MOESM3_ESM.pdf]

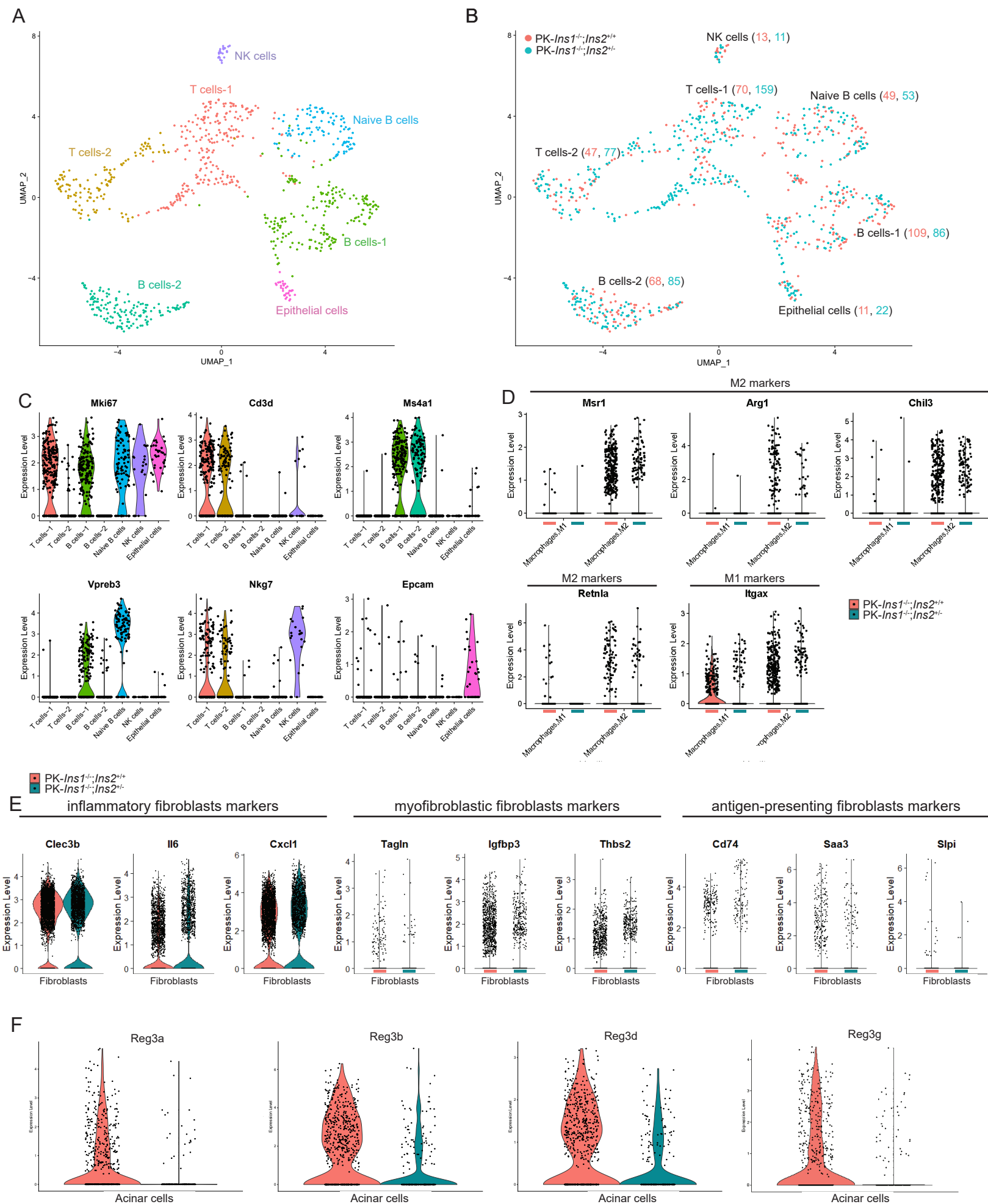

Supplemental Figure 2

Supplement: Supplementary file 4 — Additional file 4: Fig. S2. scRNAseq analysis shows there are multiple cell types in the proliferating cell cluster and there are 3 types of fibroblasts. A Unsupervised sub-clustering of the cluster containing proliferating cells, represented as an UMAP plot. The proliferating cell cluster contains proliferating T cells, B cells, Naïve B cells, NK cells and epithelial cells. B Numbers of cells from PK-Ins1-/-;Ins2+/+ (orange) and PK-Ins1-/-;Ins2+/- (green) mice for each cell type. C Violin plot showing the expression level of selected cell type-specific markers for identified cell types within the proliferating cell cluster. D Expression level of the typical markers for identifying M1 macrophages and M2 macrophages for each genotype. E Violin plots showing the expression level of selected markers for inflammatory, myofibroblastic and antigen-presenting fibroblasts. F The differential expression of Reg3a, Reg3b, Reg3d, and Reg3g genes in acinar cells between PK-Ins1-/-;Ins2+/+ and PK-Ins1-/-;Ins2+/- mice. [file 40170_2022_282_MOESM4_ESM.pdf]

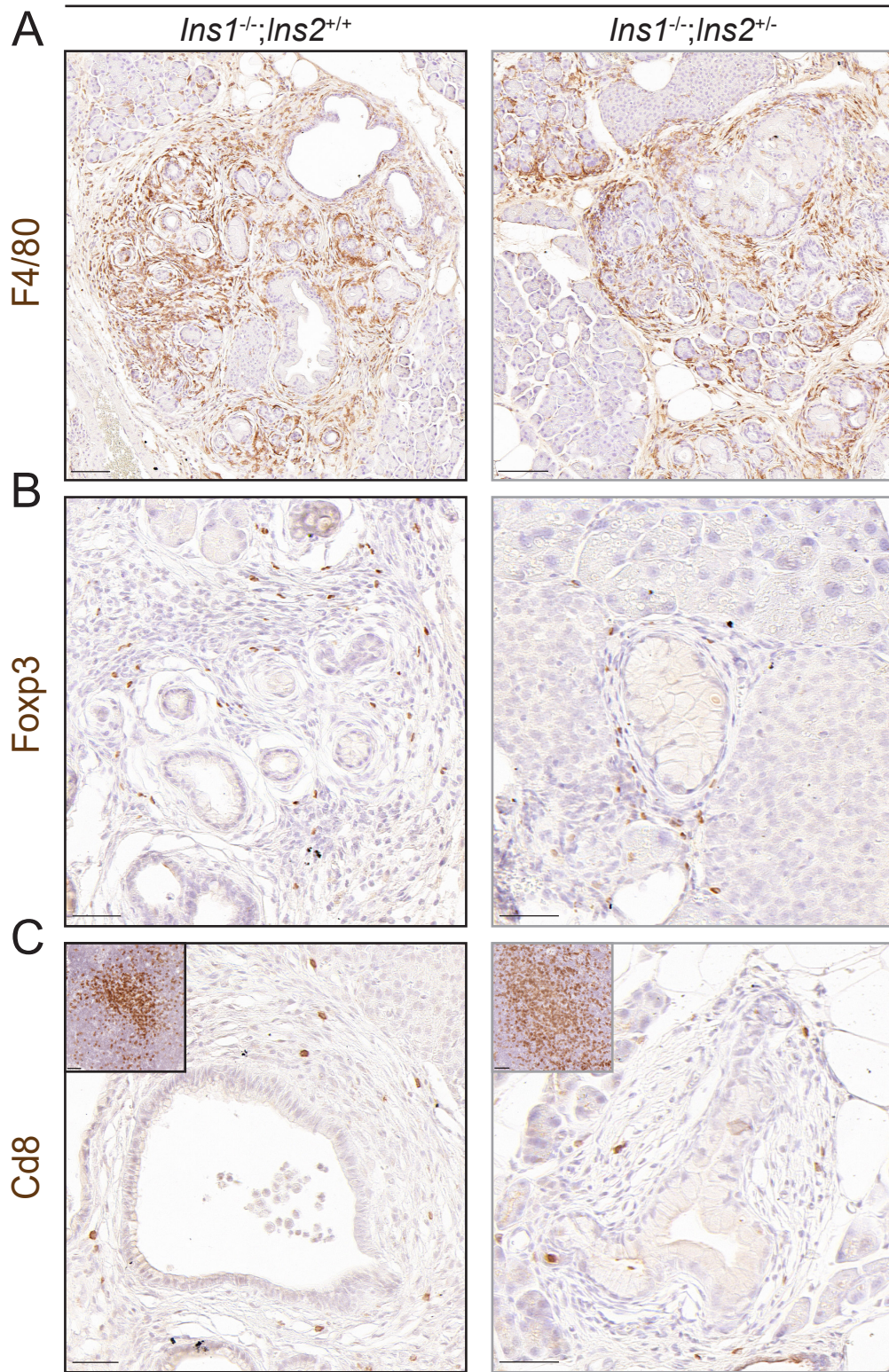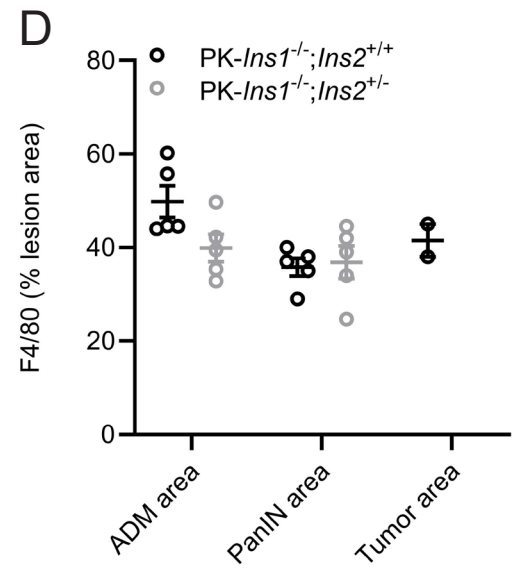

Supplement: Supplementary file 5 — Additional file 5: Fig. S3. Multiple immune cells present around PanIN and ADM lesions. A-C Immunohistochemistry of F4/80 (A), Foxp3 (B), and Cd8 (C) for PK-Ins1-/-;Ins2+/+ and PK-Ins1-/-;Ins2+/- pancreata. (Representative Cd8+ T cells in lymph nodes, C inset). D Quantification of F4/80 positive area per lesion area for each ADM, PanIN and tumor area. No PK-Ins1-/-;Ins2+/- mice developed PDAC. (A: Scale bars: 0.1mm. B-C: Scale bars: 0.05mm). [file 40170_2022_282_MOESM5_ESM.pdf]
